# Retraction: Serum amyloid a induces M2b-like macrophage polarization during liver inflammation

**DOI:** 10.18632/oncotarget.28498

**Published:** 2023-10-04

**Authors:** Yibin Wang, Haijun Huang, Renhua Sun, Bingyu Chen, Fang Han, Qian Li, Yin Ni, Xi Li, Jingquan Liu, Xiaozhou Mou, Yuexing Tu

**Affiliations:** ^1^Department of Cardiology, Chunan First People’s Hospital, Hangzhou 311700, China; ^2^Department of Infectious Diseases, Zhejiang Provincial People’s Hospital, People’s Hospital of Hangzhou Medical College, Hangzhou 310014, China; ^3^ICU Department, Zhejiang Provincial People’s Hospital, People’s Hospital of Hangzhou Medical College, Hangzhou 310014, China; ^4^Centre of Laboratory Medicine, Chunan First People’s Hospital, Hangzhou 311700, China; ^5^Department of Transfusion Medicine, Zhejiang Provincial People’s Hospital, People’s Hospital of Hangzhou Medical College, Hangzhou 310014, China; ^6^Centre of Laboratory Medicine, Zhejiang Provincial People’s Hospital, People’s Hospital of Hangzhou Medical College, Hangzhou 310014, China; ^7^Clinical Research Institute, Zhejiang Provincial People’s Hospital, People’s Hospital of Hangzhou Medicine College, Hangzhou 310014, China; ^8^Key Laboratory of Tumor Molecular Diagnosis and Individualized Medicine of Zhejiang Province, Hangzhou 310014, China; ^*^These authors have contributed equally to this work

**This article has been retracted:** Due to defects in the experimental results and methods, the conclusions of this paper are invalid. The authors were unable to verify or replicate the experiments. As a result, with the agreement of all authors, Oncotarget has decided to retract this paper.

Original article: Oncotarget. 2017; 8:109238–109246. 109238-109246. https://doi.org/10.18632/oncotarget.22652

